# Random Coil to Globular Thermal Response of a Protein (H3.1) with Three Knowledge-Based Coarse-Grained Potentials

**DOI:** 10.1371/journal.pone.0049352

**Published:** 2012-11-14

**Authors:** Ras B. Pandey, Barry L. Farmer

**Affiliations:** 1 Department of Physics and Astronomy, University of Southern Mississippi, Hattiesburg, Missouri, United States of America; 2 Materials and Manufacturing Directorate, Air Force Research Laboratory, Wright Patterson Air Force Base, Ohio, United States of America; Russian Academy of Sciences, Institute for Biological Instrumentation, Russian Federation

## Abstract

The effect of temperature on the conformation of a histone (H3.1) is studied by a coarse-grained Monte Carlo simulation based on three knowledge-based contact potentials (MJ, BT, BFKV). Despite unique energy and mobility profiles of its residues, the histone H3.1 undergoes a systematic (possibly continuous) structural transition from a random coil to a globular conformation on reducing the temperature. The range over which such a systematic response in variation of the radius of gyration (*R_g_*) with the temperature (*T*) occurs, however, depends on the potential, i.e. *ΔT_MJ_ ≈ 0.013–0.020*, *ΔT_BT_ ≈ 0.018–0.026*, and *ΔT_BFKV_ ≈ 0.006–0.013* (in reduced unit). Unlike MJ and BT potentials, results from the BFKV potential show an anomaly where the magnitude of *R_g_* decreases on raising the temperature in a range *ΔT_A_ ≈ 0.015–0.018* before reaching its steady-state random coil configuration. Scaling of the structure factor, *S(q) ∝ q^−1/ν^*, with the wave vector, *q = 2π/λ*, and the wavelength, *λ*, reveals a systematic change in the effective dimension (*D_e_∼1/ν*) of the histone with all potentials (MJ, BT, BFKV): *D_e_∼3* in the globular structure with *D_e_∼2* for the random coil. Reproducibility of the general yet unique (monotonic) structural transition of the protein H3.1 with the temperature (in contrast to non-monotonic structural response of a similar but different protein H2AX) with three interaction sets shows that the knowledge-based contact potential is viable tool to investigate structural response of proteins. Caution should be exercise with the quantitative comparisons due to differences in transition regimes with these interactions.

## Introduction

The structures of proteins have been a subject of extensive investigation for decades particularly using computer simulations (with overwhelming amount of literature, the list is too large to cite) [1]–[35]. Accurate potentials based on the structural details of atoms, molecules, and amino acids are of particular interest in modeling proteins. Incorporation of good potentials or force fields based on the fundamental interaction is highly desirable particularly in probing the structural details at small scales. With a well-defined force field based on the basic interactions (involving few fundamental parameters), it is easier to implement standard tools of statistical mechanics (e.g., molecular dynamics, Monte Carlo and variants). As a result, it is feasible to probe the effects of thermodynamic parameters such as temperature, concentration of solvent, molecular weight, substrate, etc. on the structure of protein. Due to the enormity of the time scale, it is not feasible to incorporate such elaborate force fields (involving electronic structures at atomic scales) in probing the conformational ensemble of larger proteins that can undergo dramatic structural transformation.

In order to carry out large-scale computer simulations and draw meaningful conclusions, some degree of approximation is unavoidable in almost all models involving all-atom details to minimalist coarse-grained descriptions. Some of the approximations and coarse-graining procedures include devising interaction potentials, exploring the phase space selectively, resorting to efficient and effective methods, etc. A considerable part of phenomenological modeling of proteins relies on the native structure, which is critical in performing its major function. Interaction among the amino acids (AAs), arising from fundamental atomic interactions and covalent bonding, and with the underlying matrix is critical in understanding the structure of the protein. What distinguishes one protein from another is the size of the protein (number of AAs) and the sequence. In many investigations, simplified phenomenological energy functions are considered to explain the native structure as the minimum energy state. The lowest energy configuration may not be the most probable configuration due to frustration caused by the competing effects of steric constraints (covalent bonding), interactions and temperature.

One approach used extensively to understand the structure of the protein involves residue-residue interactions based on the contact matrix, which is derived from an ensemble of frozen structures of protein available at the protein data bank (PDB) using a number of assumptions and approximations. Early knowledge-based interaction potential proposed by Tanaka and Scheraga^1^ was further developed by Miyazawa and Jernigan (MJ) [2], [3] in a mean-field spirit of an effective medium.

Over the years, a number of knowledge-based contact potentials [1]–[11] have been re-examined and redeveloped to understand the folding dynamics of a protein. For example, Betancourt and Thirumalai [7] have examined the MJ contact matrix and the potential matrix by Skolnick et al. [11] and proposed their own contact potential matrix (BT). By selecting an appropriate reference solvent (Thr) within the Miyazawa and Jernigan scheme [2], [3], they find [7] that the BT interaction matrix gives ‘hydrophobicities that are in very good agreement with experiment.’ Bastolla et al. (BFKV) [8] have examined some of these knowledge-based interaction potentials and presented a scheme to guarantee optimal stability for most representative structures. They have pointed [8] out that ‘the optimized energy function guarantees high stability and a well-correlated energy landscape to most representative proteins in the PDB database.’ We have recently implemented [33] the classical MJ interaction matrix to examine the thermal response of two histones (H3.1, H2AX) [36], [37]. These proteins are of comparable size (with 136 and 143 residues, respectively) but respond very differently to temperature. Whereas H3.1 exhibits a systematic transition from a random coil to globular conformation (see below), H2AX shows non-monotonic dependence (expanded conformations followed by compact structures) with a maximum as a function of temperature [33]. Because of the phenomenological nature of the knowledge-based interactions, it is worth re-analyzing the thermal response again with the tested and improved potentials such as BT [7] and BFKV [8] potentials in addition to classical MJ potential to understand similarity and differences in the conformational response to temperature.

In context to extracting the optimal weight associated with the knowledge-based residue-residue contact matrix elements, Pokarowski et al [35] have analyzed 29 contact potentials and concluded that ‘one-body approximations of the contact potentials could be useful in some applications.’ They have pointed out the ‘opportunities’ to develop different further types of potentials (perhaps multibody)’. Such an extensive analysis clearly underscores the fact that the knowledge-based contact potentials are phenomenological measures and are somewhat adhoc and that the reliability of results about the general features and specific response should be carefully examined. In absence of comprehensive analysis based on fundamental hierarchical interactions, knowledge-based interaction do provide a feasible mechanism to incorporate some specificity of residues in understanding the structure of a protein. We focus here on the conformation of histone H3.1 [36], [37] as a function of temperature. In order to identify common results (reproducible by different potentials) and differences, we use three contact matrices as an input to a phenomenological potential (see below) to investigate the effect of temperature on the conformation of histone H3.1:

**^1^M ^2^A ^3^R ^4^T ^5^K ^6^Q ^7^T ^8^A ^9^R ^10^ K ^11^S ^12^T ^13^G ^14^G ^15^K ^16^A ^17^P ^18^R ^19^K ^20^Q ^21^L ^22^A ^23^T ^24^K ^25^A ^26^A ^27^R ^28^K ^29^S ^30^A ^31^P ^32^A ^33^T ^34^G ^35^G ^36^V ^37^K ^38^K ^39^P ^40^H ^41^R ^42^Y ^43^R ^44^P ^45^G ^46^T ^47^V ^48^A ^49^L ^50^R ^51^E ^52^I ^53^R ^54^R ^55^Y ^56^Q ^57^K ^58^S ^59^T ^60^E ^61^L ^62^L ^63^I ^64^R ^65^K ^66^L ^67^P ^68^F ^69^Q ^70^R ^71^L ^72^V ^73^R ^74^E ^75^I ^76^A ^77^Q ^78^D ^79^F ^80^K ^81^T ^82^D ^83^L ^84^R ^85^F ^86^Q ^87^S ^88^S ^89^A ^90^V ^91^M ^92^A ^93^L ^94^Q ^95^E ^96^A ^97^C ^98^E ^99^A ^100^Y ^101^L ^102^V ^103^G ^104^L ^105^F ^106^E ^107^D ^108^T ^109^N ^110^L ^111^C ^112^A ^113^I ^114^H ^115^A ^116^K ^117^R ^118^V ^119^T ^120^I ^121^M ^122^P ^123^K ^124^D ^125^I ^126^Q ^127^L ^128^A ^129^R ^130^R ^131^I ^132^R ^133^G ^134^E ^135^R ^136^A.**

## Model and Methods

The histone H3.1 consists of *136* residues in a unique sequence [36], [37]. It is represented [31]–[33] by 136 nodes tethered together by the fluctuating bonds [38] on a cubic lattice in our coarse-grained description. A node (residue) is represented by a unit cubic cell (occupying its eight lattice sites) with excluded volume constraints [39]. The bond length between consecutive nodes can vary between *2* and *√10*. Such a bond fluctuation description is known to capture the computational efficiency while incorporating ample degrees of freedom and extensively used in complex polymer systems [39], multi-component nano-composites [39], [40] and protein chains [31]–[33], [41]. Note that the lattice model with fluctuating (i.e., expanding and contracting) covalent bonds between consecutive residues has more degrees of freedom that the minimalist HP model used for sensitivity test by Betancourt and Thirumalai [7]. The large-scale simulations become feasible with such simplified coarse-grained representation of a residue without the all-atom details while capturing the specificity of each residue via residue-residue interactions. Each residue interacts with the neighboring residues within a range (*r_c_*) with a generalized Lennard-Jones potential,
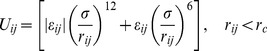
where *r_ij_* is the distance between the residues at site *i* and *j*; *r_c_ = √8* and *σ = 1* in units of lattice constant. The potential strength *ε_ij_* is unique for each interaction pair with appropriate positive (repulsive) and negative (attractive) values (see below).

The Metropolis algorithm is used to move each tethered residue randomly. For example, a residue, for instance, at a site *i*, is selected randomly to move to a neighboring lattice site, *j*. As long as the excluded volume constraints and the limitations on changes in the covalent bond length are satisfied, the residue is moved from site *i* to site *j* with the Boltzmann probability *exp(−ΔE_ij_/T)*, where *ΔE_ij_ = E_j_ – E_i_* is the change in energy between its new (*E_j_*) and old (*E_i_*) configuration and *T* is the temperature in reduced units of the Boltzmann constant and the energy (*ε_ij_*). A unit Monte Carlo step (MCS) is defined as attempts to move each residue once. During the course of simulation, we keep track of a number of local and global physical quantities including energy of each residue, its mobility, mean square displacement of the center of mass of the protein, radius of gyration and its structure factor. Simulations at each temperature are performed for a sufficiently long time (typically for ten million time steps) with many independent samples (typically 150 samples for long runs and 1000 samples for short runs) to estimate these quantities. The data presented here are generated on a 64*^3^* lattice although different lattice sizes are used to assure that there is no finite size effect on the qualitative variations of the physical quantities and our conclusions.

**Figure 1:**
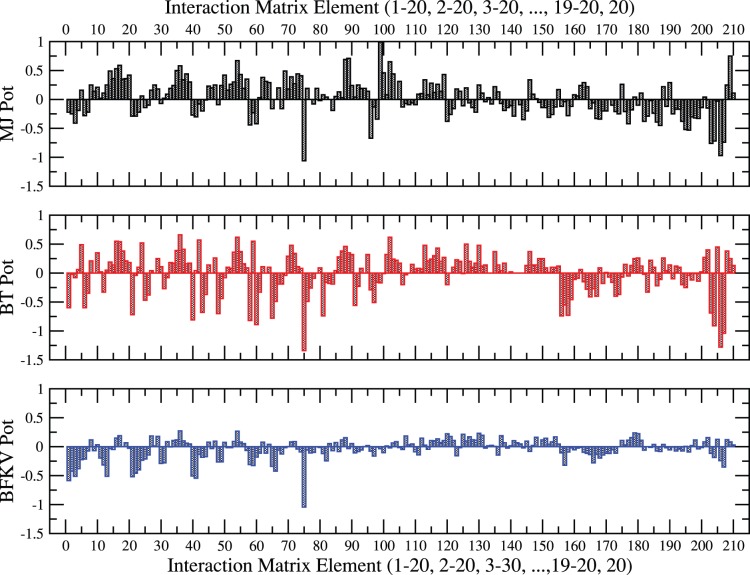
Residue-residue interaction matrix elements of MJ,^2^ BT,^7^ and BFKV.^8^ The matrix elements are labeled *1–210*: *ε_1,1_*, *ε_1,2_*, …, *ε_1,20_*, *ε_2,2_*, *ε_2,3_*, …, *ε_2,20_*, …, *ε_20,20_* based on hydropathy index ^1^I ^2^V ^3^L ^4^F ^5^C ^6^M ^7^A ^8^G ^9^T ^10^S ^11^W ^12^Y ^13^P ^14^H ^15^Q ^16^ N ^17^D ^18^ E ^19^K ^20^R.

As mentioned above, we use three knowledge-based contact matrices (figure 1), i.e., the classical MJ [2], BT [7], and BFKV [8] for the residue-residue pair interaction (*ε_ij_*). On a first look, these matrices appear somewhat similar in general apart from the difference in magnitudes. There are however differences that are easier to spot with a closer look, e.g. elements *1–10*, *190–200*, etc., which may show in the final results on the thermal response (see below).

## Results

**Figure 2:**
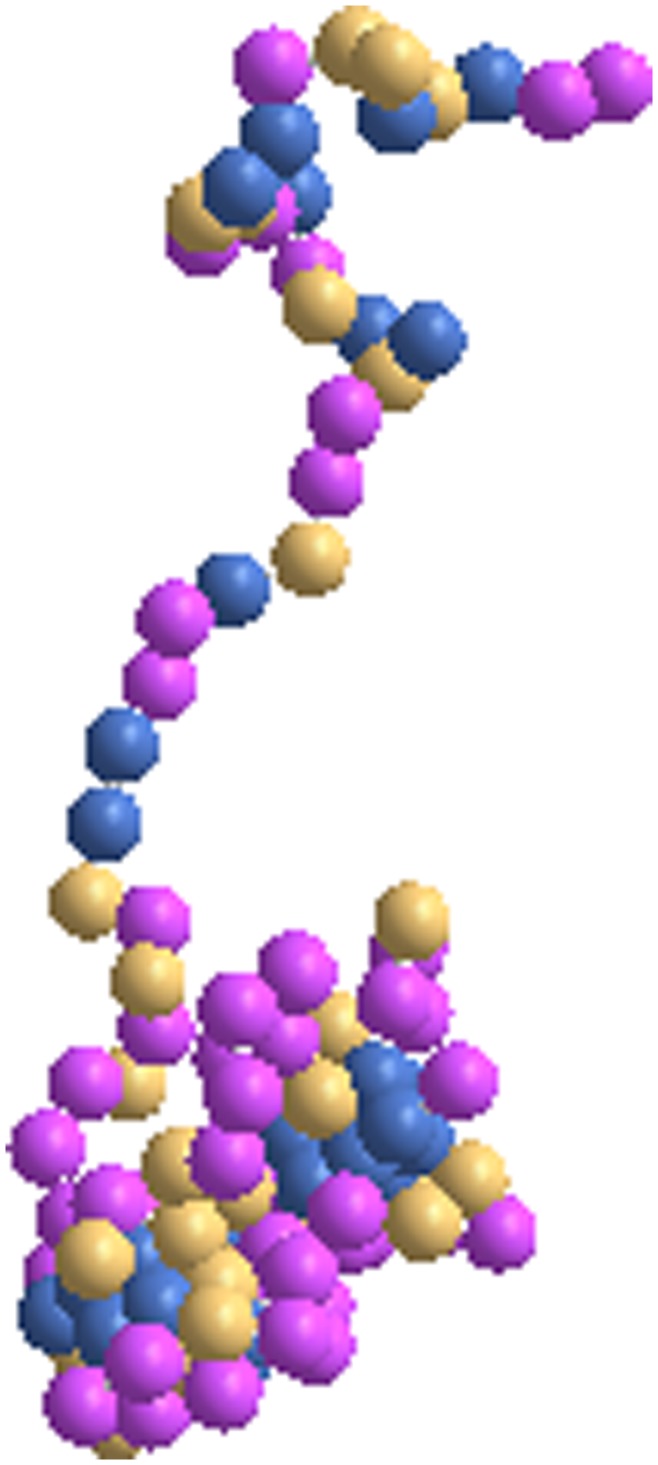
Snapshots of the histone H3.1 at *t = 10^7^* time step at the temperatures *T = 0.*016 using MJ potential^2^.

Some insight into the structural relaxations and equilibration of the conformation at local and global scales of protein can be gained from the snapshots and animations during the course of simulation. For example, see a typical snapshot of the histone at a representative temperature resulting from MJ interaction in Figure 2 at the end of the simulation, i.e., at *t = 10^7^* steps.

Note that the conformation of the protein undergoes numerous configurations (better seen in animations) and each of the snapshots is an instantaneous configuration. At low temperature (*T = 0.010*), most of the residues are organized into a compact globular conformation which opens up as the temperature is increased while maintaining some degree of local assembly. The overall size of the protein increases as the temperature increases. Which sections of the local segments coagulate while others elongate depend on the specific residues in the sequence in corresponding segments. The interactions among the residues compete with the thermal energy and this competition leads to a vast ensemble of configurations. The ensemble averaging of the local and global physical quantities provides us the trend in variation of the equilibrium structure and size of the protein as a function of the temperature.

**Figure 3:**
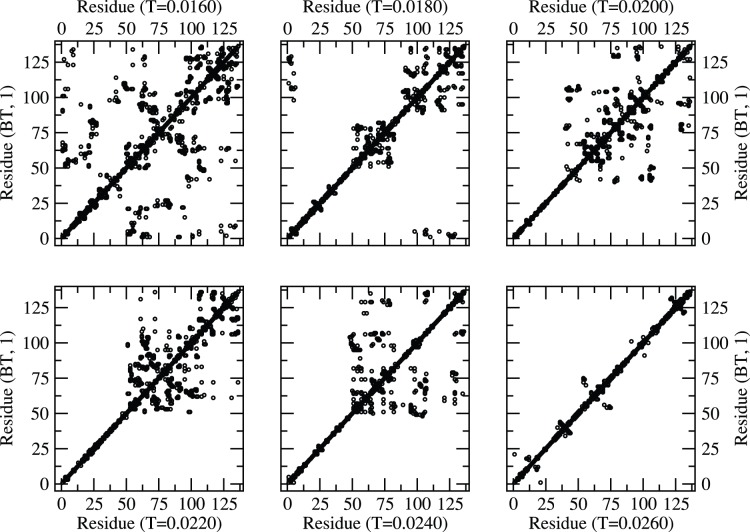
Residue map (neighboring residues along the contour within the range of interaction) of protein at different temperatures (T = 0.0160–0.0260) with BT potential.

**Figure 4:**
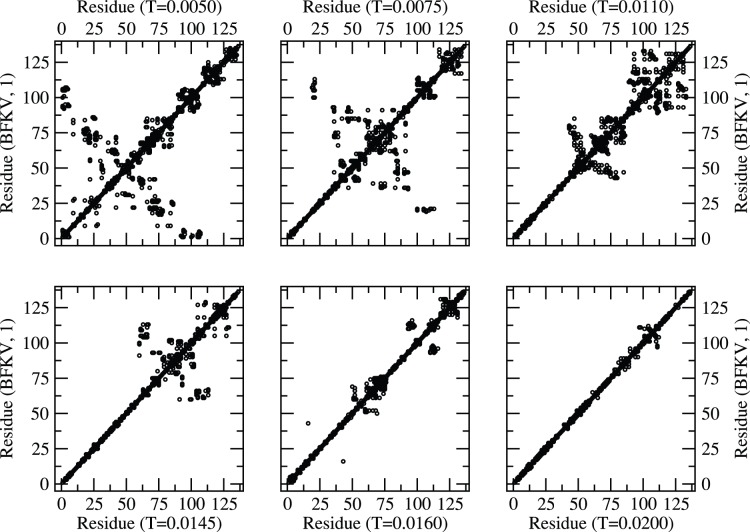
Residue map (neighboring residues along the contour within the range of interaction) of protein at different temperatures (T = 0.0050–0.0200) with BFKV potential.

The residue map of the protein structures at representative temperatures (low to high on a relative basis) with BT and BFKV potentials are presented in figures 3 and 4 respectively. Despite the difference in range of temperature in BT and BFKV schemes, we see a general systematic conformational crossover, i.e. from a compact to an elongated conformation on raising the temperature. Thus the visual inspections of the snapshots as a result of three potentials (MJ, BT, BFKV) lead to a general feature of histone H3.1, that it opens up on raising the temperature and collapses into a compact form on lowering the temperature. This may not appear dramatic, but it is particularly noteworthy that the residues in a specific sequence in H3.1 experience a diverse range of interactions but respond collectively in such an organized fashion to bring about a systematic change in global conformation (somewhat similar to homo-polymers). However, another histone (H2AX) of comparable size exhibits non-monotonic structural response [33] where the competing and cooperative effect of interacting residue leads to a very different result.

**Figure 5:**
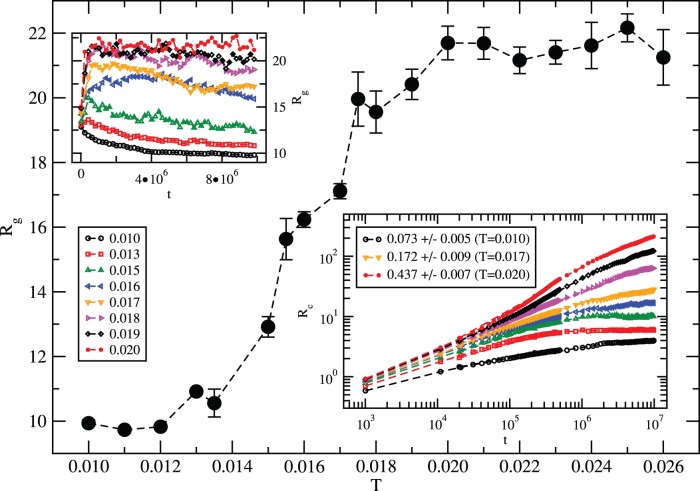
Variation of the average radius of gyration (*R_g_*) of histone H3.1 with temperature using MJ potential. Simulations are performed for *t = 10^7^* MCS time on a *64^3^* lattice with *150* independent samples; 1000 samples are used with shorter runs. Insets: top left: *R_g_* versus *t*; bottom right: RMS displacement (*R_c_*) versus *t* on a log-log scale with asymptotic slopes at *T = 0.010, 0.017, 0.020*.

Variation of the average radius of gyration (*R_g_*) with the temperature is presented in Figure 5 as a result of the MJ potential. Despite fluctuations in the data points, a systematic variation of *R_g_* with temperature seems to suggest a rather smooth transition around *T_c_∼0.013–0.015* from an extended structure at high temperature (*T≥0.020*) to a compact morphology at the low temperature (*T≤0.013*). The dependence of the root mean square (RMS) displacement (*R_c_*) of the center of mass of the protein with the time steps (*t*) also exhibits a systematic change in the global dynamics of the protein characterized by a power-law *R_c_∝t^k^*. For example, it changes from a nearly standstill (frozen-in) state (*k→0*) at low temperatures (*T≤0.013*) to a highly mobile protein with diffusive motion (*k = 1/2*) at high temperatures with a range of sub-diffusive (*1/2<k<0*) dynamics at the intermediate temperatures in the transition regime.

Regardless of appreciable fluctuations, the radius of gyration seems to reach steady-state values at most temperatures (inset figure 5). It should be pointed out that MC step (*t*) is not the real time but provides a mean to test stochastic dynamics. We also know that the asymptotic dynamics of a chain are diffusive at high temperature as for a gas molecule in a dilute gas. The approach to diffusive motion of the protein chain at high temperature is clearly seen (the inset in figure 5 for *R_c_*). Reducing the temperature leads to slow dynamics; a systematic slowing down of the protein dynamics as observed here (figure 5 inset) is thus consistent with the expectation. These trends support the reliability of such a coarse-grained approach in gaining the global insight into the structural evolution of the protein at large scales. It should be pointed out that not every sequence of *136* amino acids can lead to such transition from a random coil to a globular conformation. As mentioned above, a similar histone (H2AX) of comparable size exhibits [33] very different thermal response, i.e., a non-monotonic dependence of its gyration radius with the temperature with the same MJ potential.

**Figure 6:**
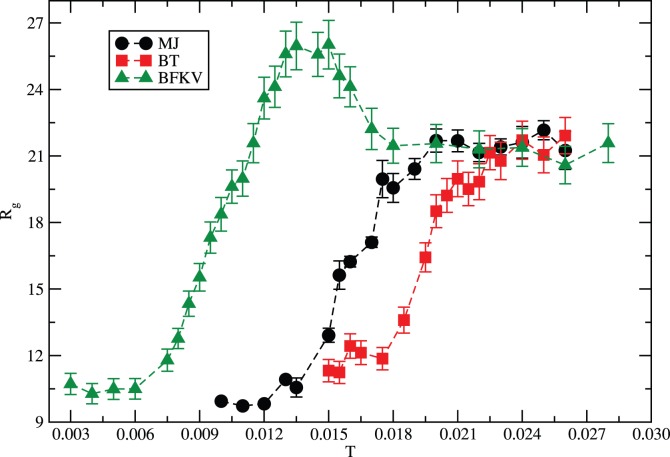
Variation of the average radius of gyration (*R_g_*) of histone H3.1 with the temperature using MJ, BT and BFKV potentials with the same statistics as in **figure 5**.

The thermal response of the radius of gyration of the protein with all three potentials, i.e., MJ, BT, and BFKV, is presented in figure 6 for comparison. We see both similarity and differences. The protein expands on raising the temperature within a range (*ΔT*), a general cooperative characteristics of the histone H3.1 results from all potentials. The range over which a systematic (monotonic) response occurs, however, depends on the potential matrix. The range of temperatures is *ΔT_MJ_≈0.013–0.020* with the MJ potential. The range shifts towards higher temperatures *ΔT_BT_≈0.018–0.026* with the BT potential and towards lower temperatures *ΔT_BFKV_≈0.006–0.013* with the BFKV potential. At the high temperature regime, the magnitudes of *R_g_* converge to a steady-state value with a random coil structure (see below) with all potentials. There is an anomaly, however, with the BFKV potential where the magnitude of *R_g_* decreases on raising the temperature in a range *ΔT_A_≈0.015–0.018* before reaching its saturation. It is difficult to know which potential is better than another over the entire range of temperatures due to lack of explicit experimental data on the variation of the *R_g_* of the histone H3.1 with the temperature. The potential matrices BT and BFKV proposed by Betancourt and Thirumalai [7] and Bastolla et al. [8], respectively, seem to be an improvement over the classical MJ potential [2]. Results from both potentials, BT and BFKV, show opposite shift with respect to MJ potential. Despite similar statistics regarding the sampling, the data for *R_g_* in figure 6 with BFKV appear smoother than that with MJ and BT which is primarily due to differences in its contact matrices.

The reduced unit of temperature (with Boltzmann constant and interaction energy) used here appears somewhat arbitrary but it is kept the same for all potential matrices in our coarse-grained MC approach as are the potentials which are phenomenological as described above. Therefore, some guidance will be welcome from clean experiments to identify the thermal response of histone H3.1. Such a calibration may help identify the range of validity or reliability of different potentials. The common features (e.g., random coil to globular transition) in thermal response resulting from all potentials may also help with understanding and interpreting the universal characteristics of the histone H3.1 in future experiments.

**Figure 7:**
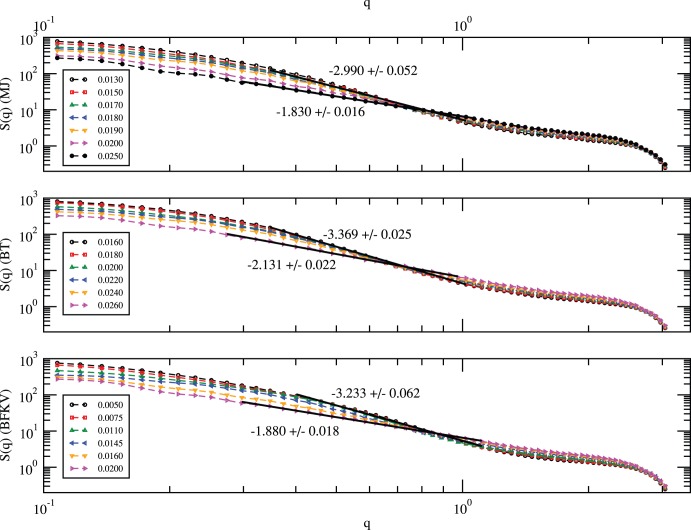
Variation of the structure factor, *S(q)*, of histone H3.1 with the wave vector, *q* with MJ, BT, and BFKV potentials from simulations on a *64^3^* lattice with *150* independent samples with *t = 10^7^* MCS time.

In order to examine the spatial distribution of residues, i.e., the shape of the protein, we have analyzed the structure factor *S(q)* (Figure 7):
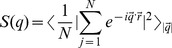
where *r_j_* is the position of each residue and *|q| = 2π/λ* is the wave vector of wavelength, *λ*. From the power-law scaling of the structure factor with the wave vector, *S(q)∝q^−1/ν^*, one can estimate the spatial distribution of residues in the protein (*R_g_ ∝ N^ν^*). Of particular interest is the range of the wave vector, *q ≈ 0.35–0.75* corresponding to the average size of the protein, *R_g_≈10–26* (see figure 6). The estimate of the effective spatial dimension (*D_e_ ≈ 1/ν*) of the protein with the MJ potential leads to *D_e_≈2*, a random coil (ideal chain) at the high temperature *T = 0.025*, and *D_e_≈3*, a solid globular structure (*ν = 1/3*) at the low temperature *T = 0.013*. Similar scaling fits are also consistent with the results of BT and BFKV potentials (figure 7). Note that at high temperatures, thermal energy dominates over residue-residue interaction. Therefore, the protein chain behaves like an ideal polymer chain (with excluded volume constraints) as it should.

## Discussion and Conclusions

A coarse-grained Monte Carlo simulation is used to investigate the effect of temperature on the conformation of a protein, histone H3.1, using three knowledge-based potentials, the classical MJ, BT and BFKV. The protein is described by a coarse-grained chain of residues (nodes) tethered together by fluctuating covalent bonds. Each residue interacts with other residues within a range of interaction using a generalized LJ potential where knowledge-based contact matrices (MJ, BT, BFKV) are used as an input for the residue-residue interaction. The coarse-grained interaction matrix thus captures the specificity of each residue. Extensive simulations are performed for a range of temperatures for sufficiently long time steps to identify changes in conformation and stability. We have examined a number of local and global physical quantities including the energy of each residue, its mobility, mean square displacement of the center of the mass of the protein, radius of gyration and its structure factor. Thermal responses resulting from the three potentials are compared and similarities and differences are pointed out.

Global conformation (measured by the radius of gyration and the structure factor) resulting from the collective dynamics of residues in histone H3.1 depends on temperature. How it changes depends on the interaction potential and the range of temperature. One of the general characteristics common to results from all three potentials (MJ, BT, BFKV) is that the protein undergoes a systematic conformational transformation: globular conformation at low temperature to a random coil at high temperatures (simple but unique to H3.1). The range over which such a systematic response occurs, however, depends on the potential matrix. For example, *ΔT_MJ_ ≈ 0.013–0.020* with the MJ potential, and shifts towards higher temperatures *ΔT_BT_ ≈ 0.018–0.026* with the BT potential and lower temperatures *ΔT_BFKV_ ≈ 0.006–0.013* with the BFKV potential. The magnitudes of *R_g_* converge to a steady-state value with a random coil structure with all potentials in the high temperature regime. Variation of *R_g_* with the temperature shows an anomaly with the BFKV potential where the magnitude of *R_g_* decreases on raising the temperature in a range *ΔT_A_ ≈ 0.015–0.018* before reaching its saturation. Despite the improved potentials suggested by both groups, Betancourt and Thirumalai [7] and Bastolla et al. [8], it is not clear which potential is better than the other over the entire range of temperatures. Results from both potentials, BT and BFKV, show opposite shift with respect to the MJ potential. One of the main problems is the lack of explicit experimental data on the variation of the *R_g_* of the histone H3.1 with temperature.

The power-law scaling of the structure factor with the wave vector, *S(q) ∝ q^−1/ν^*, a consequence of the spatial distribution of residues (*R_g_ ∝ N^ν^* with *N* being the number of residues) in the protein provides an insight into the overall morphology. The wave vector in the range of the radius of gyration leads to an effective dimension *D_e_ ≈ 1/ν* of protein. Results of all three potentials (MJ, BT, BFKV) are consistent with our assessment about the global structure of the protein, i.e., the globular conformation *D_e_≈3* in low temperature regime and a random coil (ideal chain) *D_e_≈2* at the high temperatures.

Because of the unique sequence of interacting residues, the segmental morphology is heterogeneous and shows enormous variability. The cooperative and competing effect of these segments is expected to exhibit complex temperature dependence as seen with other histones [33]. It is remarkable to observe such a continuous global transformation from a random coil to a globular structure on cooling. The cooperative assembly of residues seems to propagate on larger (genetic) length scales smoothly despite a rather random but unique distribution of attractive and repulsive segments (residues) of the protein. The structural response of the histone H3.1 to temperature is very different from that of the histone H2AX, which shows non-linear (non-monotonic) thermal response [33]. It must be pointed out that the histone H3.1 plays a critical role in response to the cell’s cycle in the structural response of DNA in translation, transcription, and replication while the histone H2AX is believed to be crucial in mounting the response to repairing damaged DNA [42]–[48]. Therefore, the differences in thermal response of the global structures of two different histones seem appropriate for performing the distinct functions of each histone.

A protein can thus undergo a continuous conformational transition. Why is such a systematic thermal response in the structure of a protein important? First, to our knowledge, it is not common in such a complex protein. Second, it provides insight into the global response with respect to local characteristics of residues with its multi-scale hierarchical structural evolution. One may question that the size of the protein H3.1 (with 136 residues) is too small to observe a possible continuous change in conformation in thermodynamic limit (i.e., a protein chain with infinite size). While one cannot rule out such a possibility, completely different thermal response [33] (non-monotonic) of dissimilar proteins (e.g., H2AX) of comparable size (e.g., 143 residues) leads us to believe that the competition between the temperature and the characteristic interactions among the residues in a specific sequence is critical.

Coarse graining in modeling proteins is not unique; there can be many possibilities to develop alternate and new methods that may verify, complement or provide evidence against our findings. The variety of proteins and their characteristics is so huge (even in the histone family) that identifying their common characteristics is not feasible but highly desirable. If many proteins are found to exhibit such a thermodynamic transition, then one may be able to identify their common characteristics and special features, which may help identify universality in the non-universal world of peptides and proteins.

## References

[1] Tanaka S, Scheraga HA (1976) Medium and long range interaction parameters between amino acids for predicting three dimensional structures of proteins. Macromolecules 9: 945–950. 1004017 10.1021/ma60054a013

[2] Miyazawa S, Jernigan RL (1985) Estimation of effective interresidue contact energies from protein crystal structures: quasi-chemical approximation. Macromolecules 18: 534–552.

[3] Miyazawa S, Jernigan RL (1996) Residue-residue potentials with a favorable contact pair term for simulation and treading. J Mol Biol 256: 623–644. 8604144 10.1006/jmbi.1996.0114

[4] Residue-residue interaction tables website, courtesy of the research group of R.L. Jernigan. Available: http://gor.bb.iastate.edu/potential/. Accessed 2012 Oct 10.

[5] Bagci Z, Kloczkowski A, Jernigan RL, Bahar I (2003) The Origin and Extent of Coarse Grained Irregularities in Protein Internal Packing I. Proteins. 53: 56–67. 10.1002/prot.10435 12945049

[6] Liwo A, Pincus MR, Wawak RJ, Rackovsky S, Scheraga HA (1993) Calculation of protein backbone geometry from alpha-carbon coordinates based on peptide-group dipole alignment. Prot Sci 2: 1697–1714. 10.1002/pro.5560021015 PMC2142257 7504550

[7] Betancourt MR, Thirumalai D (1999) Pair potentials for protein folding: choice of reference states and sensitivity of predicted native states to variations in the interaction schemes. Protein Sci 2: 361–369. 10.1110/ps.8.2.361 PMC2144252 10048329

[8] Bastolla U, Farwer J, Knapp EW, Vendruscolo M (2001) How to guarantee optimal stability for most representative structures in the proten data bank. Proteins 44: 79–96. 11391771 10.1002/prot.1075

[9] Maiorov VN, Crippen GM (1992) Contact potential that recognizes the correct folding of globular proteins. J Mol Biol 227: 876–888. 1404392 10.1016/0022-2836(92)90228-c

[10] Godzik A, Kolinski A, Skolnick J (1996) Knowledge-based potentials for protein folding: what can we learn from protein structures? Proteins 4: 363–366. 10.1016/s0969-2126(96)00041-x 8740358

[11] Skolnick J, Jaroszewski L, Kolinski A (1997) Derivation and testing of pair potentials for protein folding: When is the quasichemical approximation correct? Protein Sci 6: 676–688. 9070450 10.1002/pro.5560060317 PMC2143667

[12] Hinds DA, Levitt M (1992) A lattice model for protein structure prediction at low resolution. Proc Natl Acad Sci USA 89: 2536–2540. 1557356 10.1073/pnas.89.7.2536 PMC48696

[13] Fiebig KM, Dill KA (1993) Protein Core Assembly Processes. J Chem Phys 98: 3475–3487.

[14] Bryant SH, Lawrence CE (1993) An empirical energy function for threading protein sequence through folding motif. Proteins 16: 92–112. 8497488 10.1002/prot.340160110

[15] Wolynes PG, Onuchic JN, Thirumalai D (1995) Navigating the folding routes. Science 267: 1619–1620. 7886447 10.1126/science.7886447

[16] Tobi D, Shafran G, Linial N, Elber R (2000) On the design and analysis of protein folding potentials. Proteins 40: 71–85. 10813832 10.1002/(sici)1097-0134(20000701)40:1<71::aid-prot90>3.0.co;2-3

[17] Vendruscolo M, Najmanovich, Domany E (2000) Can a pairwise contact potential stabilize native protein folds against decoys obtained by threading? Proteins 38: 134–148. 10656261 10.1002/(sici)1097-0134(20000201)38:2<134::aid-prot3>3.0.co;2-a

[18] Skepo M, Linse P, Arnebrant T (2006) Coarse-Grained Modeling of Proline Rich Protein 1 (PRP-1) in Bulk Solution and Adsorbed to a Negatively Charged Surface. J Phys Chem B 110: 12141–12148. 16800528 10.1021/jp056033o

[19] Gillespie B, Plaxco KW (2004) Using protein folding rates to test protein folding theories. Annu Rev Biochem 73: 837–859. 15189160 10.1146/annurev.biochem.73.011303.073904

[20] Banavar JR, Maritan A (2003) Colloquium: geometrical approach to protein folding: a tube picture. Rev Mod Phys 75: 23–34.

[21] Zhou Y, Karplus M (1999) Interpreting the folding kinetics of helical proteins. Nature 400: 400–403. 10.1038/43937 10517642

[22] Shen M, Freed KF (2002) All-atom fast protein folding simulations: the villin headpiece. Proteins 49: 439–445. 12402354 10.1002/prot.10230

[23] Kuhlman B, Dantas G, Ireton GC, Varani G, Barry L, et al (2003) Design of a Novel Globular Protein Fold with Atomic-Level Accuracy. Science 302: 1364–1368. 14631033 10.1126/science.1089427

[24] Sorin EJ, Pande VS (2005) Exploring the Helix-Coil Transition via All-Atom Equilibrium Ensemble Simulations. Biophys J 88: 2472–2493. 15665128 10.1529/biophysj.104.051938 PMC1305346

[25] Moskovitz Y, Srebnik S (2012) Thermal stability limits of proteins in solution and adsorbed on a hydrophobic surface. Phys Chem Chem Phys 14: 8013–8022. 22547225 10.1039/c2cp00005a

[26] Herges T, Wenzel W (2005) In silico folding of a three helix protein and characterization of its free-energy landscape in an all-atom force field. Phys Rev Lett 94: 018101–018104. 15698135 10.1103/PhysRevLett.94.018101

[27] Gertsman B, Garbourg Y (1998) Structural Information Content and Lyapunov Exponent Calculation in Protein Unfolding. Journal of Polymer Science B: Polymer Physics 36: 2761–2769.

[28] Chapagain PP, Gertsman B (2006) Removal of kinetic traps and enhanced protein folding by strategic substitution of amino acids in a model alpha-helical hairpin peptide. Biopolym 81: 167–178. 10.1002/bip.20388 16215990

[29] Behringer H, Degenhard A, Schmid F (2006) Coarse-Grained Lattice Model for Molecular Recognition. Phys Rev Lett 97: 128101–128104. 17026000 10.1103/PhysRevLett.97.128101

[30] Peng S, Ding F, Urbanc B, Buldyrev SV, Cruz L, et al (2004) Discrete molecular dynamics simulations of peptide aggregation. Phys Rev E 69: 041908–1-041908–7. 10.1103/PhysRevE.69.041908 15169044

[31] Pandey RB, Farmer BL (2008) Conformation of a coarse-grained protein chain (an aspartic acid protease) model in effective solvent by a bond-fluctuating Monte Carlo simulation. Phys Rev E 77: 031902–031910. 10.1103/PhysRevE.77.031902 18517417

[32] Pandey RB, Farmer BL (2010) Globular structure of a human immunodeficiency virus-1 protease (1DIFA dimer) in an effective solvent medium by a Monte Carlo simulation. J Chem Phys 132: 125101–125106. 20370150 10.1063/1.3358340

[33] Fritsche M, Pandey RB, Farmer BL, Heermann D (2012) Conformational temperature-dependent behavior of a histone h2ax: A coarse-grained Monte Carlo approach via knowledge-based interaction potentials. PLoS One 2012; 7: e32075-1 – e32075-8. 10.1371/journal.pone.0032075 PMC3307718 22442661

[34] Freddolino PL, Harrison CB, Liu Y, Schulten K (2010) Challenges in protein-folding simulations, Nature Physics. 6: 751–758. 10.1038/nphys1713 PMC3032381 21297873

[35] Pokarowski P, Kloczkowski A, Jernigan RL, Kothari NS, Pokarowska M (2005) Inferring ideal amino acid interaction forms from statistical protein contact potentials, Proteins. 59: 49–57. 10.1002/prot.20380 PMC4417612 15688450

[36] Histone H3.1 website. Available: http://en.wikipedia.org/wiki/HIST1H3A. Accessed 2012 Oct 10.

[37] Sequence details including X-ray crystallography data of histone H3.1 website. Available: http://www.uniprot.org/uniprot/P68431. Accessed 2012 Oct 10.

[38] Binder K. Monte Carlo and Molecular Dynamics Simulations in Polymer Science. New York: Oxford University Press; 1995.

[39] Pandey RB, Farmer BL (2008) Exfoliation of a stack of platelets and intercalation of polymer chains: effects of molecular weight, entanglement, and interaction with the polymer matrix. J Polym Sci B 46: 2696–2710.

[40] Pandey RB, Heinz H, Farmer BL, Drummy LF, Jones SE, et al (2010) Layer of clay platelets in a peptide matrix: binding, encapsulation, and morphology. J Polym Sc B 48: 2566–2574.

[41] Eby DM, Johnson GR, Farmer BL, Pandey RB (2011) Supramolecular assembly of a biomineralizing antimicrobial peptide in coarse-grained Monte Carlo simulations. Phys Chem Chem Phys 13, 2123–1130. 10.1039/c0cp01364a 21072418

[42] Tagami H, Ray-Gallet D, Almouzni G, Nakatani Y (2004) Histone H3.1 and H3.3 complexes mediate nucleosome assembly pathways dependent or independent of DNA synthesis. Cell 116: 51–61. 14718166 10.1016/s0092-8674(03)01064-x

[43] Tachiwana H, Osakabe A, Kimura H, Kurumizawa H (2008) Nucleosome formation with the testis-specific histone H3 variant, H3t, by human nucleosome assembly proteins in vitro. Nuceic Acids Res 36: 2208–2218. 10.1093/nar/gkn060 PMC2367731 18281699

[44] Nakayama J, Rice JC, Strahl BD, Allis CD, Grewal SI (2001) Role of histone H3 lysine 9 methylation in epigenetic control of heterochromatin assembly. Science 292: 110–113. 11283354 10.1126/science.1060118

[45] Rea S, Eisenhaber F, O’Carroll D, Strahl BD, Sun ZW, et al (2000) Regulation of chromatin structure by site-specific histone H3 methyltransferases. Nature 406: 593–599. 10949293 10.1038/35020506

[46] Cottingham K (2008) Revealing the secrets of histone H3 modification. J Proteome Res 7: 4211. 18700790 10.1021/pr800556r

[47] Strahl BD, Ohba R, Cook RG, Allis CD (1999) Methylation of histone H3 at lysine 4 is highly conserved and correlates with transcriptionally active nuclei in Tetrahymena. Proc Natl Acad Sci USA 96: 14967–14972. 10611321 10.1073/pnas.96.26.14967 PMC24756

[48] Williams SK, Truong D, Tyler JK (2008) Acetylation in globular core of histone H3 on lysine-56 promotes chromatin disassembly during transcriptional activation. Proc Natl Acad Sci USA 105: 9000–9005. 18577595 10.1073/pnas.0800057105 PMC2449354

